# KIT Mutation Incidence and Pattern of Melanoma in Central Europe

**DOI:** 10.1007/s12253-019-00788-w

**Published:** 2019-12-17

**Authors:** V. Doma, T. Barbai, M.-A. Beleaua, I. Kovalszky, E. Rásó, József Tímár

**Affiliations:** 1 Department of Dermatology, Semmelweis University, Budapest, Hungary; 2 2nd Department of Pathology, Semmelweis University, Üllői 93., Budapest, H-1091 Hungary; 3 Department of Pathology, Pharmacy and Science, University of Medicine, Tirgu-Mures, Romania; 4 1st Department of Pathology and Experimental Cancer Research, Semmelweis University, Budapest, Hungary

**Keywords:** Skin melanoma, Mucosal melanoma, KIT mutation and sanger sequencing

## Abstract

Data on the KIT mutation rate in melanoma in the central European region is missing. Accordingly, in a cohort of 79 BRAF/NRAS double wild type cutaneous melanoma and 17 mucosal melanoma KIT mutation was assessed by Sanger sequencing of exons 9,11,13,17 and 18. In this cutaneous melanoma cohort KIT mutation frequency was found to be 34/79 (43.04%) with a significantly higher rate in acrolentiginous melanoma (ALM) as compared to UV-induced common variants (20/34, 58.8% versus 14/45, 31.1%, *p* = 0.014). In the double wild type mucosal melanoma cohort the KIT mutation frequency was found to be comparable (41.2%). The actual frequency of KIT mutation in the original 227 patient cutaneous melanoma cohort was 34/227, 14.9%. Exon 11 was the most frequent mutation site (44.7%) followed by exon 9 (21.1%) equally characterizing UV-induced common histotypes and ALM tumors. In mucosal melanoma exon 9 was the most frequently involved exon followed by exon 13 and 17. KIT mutation hotspots were identified in exon 9 (c482/491/492), in exon 11 (c559,c572, c570), in exon 13 (c642), in exon 17 (c822) and in exon 18 (c853). The relatively high KIT mutation rate in cutaneous melanoma in this central-European cohort justifies regular testing of this molecular target in this entity, not only in mucosal variants.

## Introduction

KIT is the genetic driver of several malignant tumors: the majority of GIST [1] and mastocytoses [2] harbor this gene mutation and during the progression of AML, KIT mutation may also be acquired [3]. In the past decade accumulating molecular epidemiologic evidence indicated that KIT mutation also occurs in melanoma. [4]. The major driver of skin melanoma is the mutant BRAF in almost half of the cases followed by NRAS mutation as the second most frequent one [5, 6]. However, on the third place on the list of mutant oncogenes is KIT in skin melanoma, initially considered to occur in melanomas of the non-UV sites [5, 6]. Meanwhile, reports on various ethnicities and geographic areas of the world reported that the incidence rates of KIT mutation in skin melanoma are highly variable [7] but still considered very low based on the initial observations [4, 6]. Molecular epidemiology studies indicated that the mutation pattern of mucosal melanoma is fundamentally different as compared to cutaneous locasion since KIT is the most frequently involved oncogene (15–25%) followed by a much less frequent involvement of BRAF, NRAS (<10%). [8, 9]

The mutational profile of KIT in various cancer types is another issue. Exon 8 is only involved in AML and mastocytosis [2, 3], while exon 9 is a relatively rare mutation site of KIT in GIST [9] and melanoma [7]. The most frequently involved exon of KIT is exon 11 which predominates GIST [10]. However, involvement of exon 13 and 17 of KIT in mutational changes is also frequent in various cancer types. The exon mutation pattern of KIT has significance in the sensitivity to KIT inhibitors since these mutations do not necessarily result in sensitivity but also in constitutive resistance to these drugs. The purpose of this study was to asses the molecular epidemiology of KIT mutation in melanoma in a Central European country (Hungary), since data are almost absent with an exception of a Slovenian report on a small cohort [11]. Furthermore, we wanted to characterize the mutational pattern and hotspots of KIT in melanoma since the majority of studies did not provide a comprehensive picture.

## Materials and Methods

### Patient Selection

This study was carried out in strict accordance with the Declaration of Helsinki and was approved by the Semmelweis University Regional and Institutional Committee of Science and Research Ethics (IRB, SE TUKEB 114/2012). Cases were enrolled from pathological FFPE archives of the primary tumors from Semmelweis University, Budapest tested diagnostically between 2014 and 2018 for BRAF and NRAS mutations. This cutaneous melanoma cohort consisted of UV-induced variants (SSM, NM, LMM) as well as non-UV induced acrolentiginous (ALM) forms. The BRAF/NRAS double wild type part of the cohort contained 55 primary tumors and 24 metastatic cases where the primary tumor was not accessible for testing. (Table 1) For comparison, we have collected a small but similar BRAF/NRAS wild-type mucosal melanoma cohort of 17 patients dominated by females (12/17). The localisation of mucosal melanoma was typical: the oral, anal and genital forms were equally distributed (5/17, 4/17, 4/17, respectively) similar to the gastrointestinal ones (colon, esophagus, parotis, 4/17).

**Table 1:** Clinical characteristics of the skin melanoma cohort

	Whole cohort *N* = 79 (100%)		Primary tumour characteristics *n* = 55 (100%)		Metastasis characteristics *n* = 24 (100%)
Primary cutaneous melanoma	55 (70)	Site		Site of the corresponding primary melanoma	
Metastasis of cutaneous melanoma	24 (30)	Head and neck	5 (9)	Head and neck	0 (0)
Patient characteristics		Trunk	9 (16)	Trunk	3 (13)
Gender		Upper extremity	9 (16)	Upper extremity	2 (8)
Male	43 (54)	Lower extremity	32 (58)	Lower extremitx	14 (58)
Female	36 (46)	Data not available	0 (0)	Data not available	5 (21)
Site of primary cutaneous melanoma		Histological subtype		Histological subtype of the primary melanoma	
Head and neck	7 (8)	ALM	32 (58)	ALM	3 (13)
Trunk	19 (21)	non-ALM (SSM, NM, LMM, NOS)	23 (42)	non-ALM (SSM, NM, LMM, NOS)	21 (87)
Upper extremity	11 (12)	Breslow thickness (mm)		Breslow thickness (mm) of primary melanoma	
Lower extremity	47 (53)	1,00	3 (5)	1,00	3 (13)
Data not available	5 (6)	1,01-2,00	7 (13)	1,01-2,00	1 (4)
Histological subtype		2,01-4,00	16 (29)	2,01-4,00	5 (21)
ALM	35 (44))	4,00	27 (49)	4,00	7 (29)
non-ALM (SSM, NM, LMM, NOS)	44 (56)	Data not available	2 (4)	Data not available	8 (33)
Breslow thickness (mm)		Stage		Stage	
1,00	6 (7)	IA	1 (2)	IA	3 (13)
1,01-2,00	8 (10)	IB	2 (4)	IB	0 (0)
2,01-4,00	21 (27)	IIA	4 (7)	IIA	2 (8)
4,00	34 (43)	IIB	0 (0)	IIB	0 (0)
Data not available	10 (13)	IIIA	14 (25)	IIIA	2 (8)
Stage		IIIB	5 (9)	IIIB	3 (13)
IA	4 (5)	IVA	14 (25)	IVA	3 (13)
IB	2 (3)	IVB	13 (24)	IVB	4 (16)
IIA	6 (7)	Data not available	2 (4)	Data not available	7 (29)
IIB	0 (0)			Location of cutaneous melanoma metastasis	
IIIA	16 (20)			Lymph node	7 (29)
IIIB	8 (10)			Subcutaneous	8 (33)
IVA	17 (22)			Local recidive	3 (13)
IVB	17 (22)			Lung	1 (4)
Data not available	9 (11)			Gastrointestinal	4 (16)
				Data not available	1 (4)

*ALM* acrolentiginous melanoma, *LMM* lentigo maligna melanoma, *NM* nodular melanoma, *NOS* not otherwise specified, *SSM* superficial spreading melanoma

### DNA Extraction and PCR

Before DNA extraction, a section stained with H&E was prepared to label and macrodissect the optimal tumor area and to evaluate tumor/normal cell ratio under light microscope. Tumor to normal cell ratio was determined by counting nuclei at three 40× lens fields by an experienced pathologist (JT). High Pure PCR Template Preparation Kit (Roche Holding Ltd., Basel, Switzerland) was used to isolate DNA, which was quantified using NanoDrop ND-1000 UV-Vis Spectrophotometer (NanoDrop Technologies, Wilmington, DE).

PCR amplification of exon 15 with BRAF specific primers yielded a 197 base pair product. This was analysed using restriction fragment length polymorphism (RFLP) by digestion with TspRI enzyme (New England Biolabs, Ipswich, MA) to screen for codon 600 mutant BRAF. Products were separated using 3% agarose gel electrophoresis, stained by ethidium bromide and the fragments could be exactly identified based on the estimated length of the separated products. The basis of the method is that V600 mutation abolishes the restriction site resulting in a prominent band of 212 bp of the mutant allele, whereas wild type of BRAF is completely digested enzymatically, yielding DNA fragments at 125 bp. Samples bearing BRAF mutation by RFLP were evaluated by direct sequencing of the purified PCR product.

BRAF wild type samples underwent NRAS exon 2, 3 sequencing, and the double wild-type (BRAF, NRAS) samples were screened further for c-KIT exon 9, 11, 13, 17, 18 mutations. The sequencing reaction was completed with BigDye Terminator v1.1 Cycle Sequencing Kit according to the manufacturer’s protocol on a 4-capillary automated sequencer (Applied Biosystems 3130 Genetic Analyzer) using the same primers for the PCR amplification reactions. Before analysis, purification of the sequencing reaction products was performed by BigDye XTerminatorTM Purification Kit. All kits, reagents and equipments used for sequencing were purchased from Life Technologies Corporation (Carlsbad, CA). Chromas Lite Version 2.1 software was applied to detect mutations compared to NCBI (National Center for Biotechnology Information) Nucleotide BLAST (Basic Local Alignment Search Tool) Human Database. The sensitivity of detection mutant allele was determined as 15%.

### Sanger Sequencing

The primers were designed for BRAF, NRAS and c-KIT with Array Designer software (Premier Biosoft International, Palo Alto, CA) and purchased from Integrated DNA Technologies (Coralville, IA). Primer sequences were the following: BRAF exon 15 sense: 5’-TTCCTTTACTTACTACACCTCAGA-3′, BRAF exon 15 antisense: 5′-TGGAAAAAT-AGCCTCAATTC-3′, NRAS exon 2 sense: 5’-TTGCTGGTGTGAAATGACTGAG-3′, NRAS exon 2 antisense: 5’-ATATGGGTAAAGATGATCCGACAAG-3′, NRAS exon 3 sense: 5’-AAACAAGTGGTTATAGATGGTGAAAC-3′, NRAS exon 3 antisense: 5’-GTAGAGGTTAATATCCGCAAATGAC-3′, KIT9S: AAGTATGCCACATCCCAAGTG; KIT9A: GGTAGACAGAGCCTAAACATCC; KIT11S: CAGAGTGCTCTAATGACTGAGAC; KIT11A: AAGCCACTGGAGTTCCTTAAAG; KIT13S: CTTGACATCAGTTTGCCAGTTG, KIT13A: TCCAAGCAGTTTATAATCTAGCATTG; KIT17BS: AAAAGTTAGTTTTCACTCTTTACAAG, KIT17BA: CTTAATTTGACTGCTAAAATGTGTG; KIT18S: TCAGCAACAGCAGCATCTATAAG, KIT18A: CAAGGAAGCAGGACACCAATG.

We used primers at 1 μM final concentrations per reaction. Applied Biosystems AmpliTaq Gold 360 Master Mix was purchased from Life Technologies Corporation. Each reaction (25 μl in volume) contained a minimum of 200 ng DNA and was run on Swift MaxPro Thermal Cycler (ESCO Healthcare, Singapore) with the following thermal profile: (1) activation at 95 °C for 10 min, (2) amplification (38 cycles): denaturation at 95 °C for 1 min, annealing at 55 °C for 1 min, extension at 72 °C for 2 min and (3) final extension at 72 °C for 5 min. PCR products (BRAF, NRAS, c-KIT) were separated on 2% agarose gel. The band was excised and DNA purified using the EZ-10 SPIN Column DNA Gel Extraction Kit (Bio Basic Inc., NY).

## Results

In a 227 pts. of cutaneous melanoma cohort consecutively diagnostically tested between 2014 and 2018, we found that the BRAF mutation frequency in skin melanoma in Hungary is 45.4% (103/227) confirming data from other ethnicities and geographical regions. Further analysis of the 124 BRAF-wild type skin melanomas, we found the NRAS mutation frequency to be 20% (45/227). In this way we have collected a 79-case cohort of double-wild type skin melanoma, the pathological and clinical data of which are shown in Table 1. The cohort is predominated by UV-induced common variants but contained also a significant amount (one third) of non-UV induced form (ALM). The KIT mutation frequency, based on sequencing exons 9, 11, 13, 17 and 18, in this double-wild type cohort was found to be 34/79, 43.04%. It is of note that in three cases where BRAF mutation determination was repeated by a higher sensitivity pyrosequencing, we have observed double mutations of BRAF and KIT, indicating that these mutations are not necessarily mutually exclusive, or a genetic heterogeneity of these primary tumors. KIT mutation was found significantly more frequent in ALM as compared to UV-induced common variants (58.8% versus 31.1%, *p* = 0.014, Table 2)*.* Calculation of KIT mutation frequency for our entire 227 pts. skin melanoma cohort indicated a 14.9% incidence rate which corresponds to the higher ranges published worldwide. In comparison, in the double wild type mucosal melanoma cohort the KIT mutation frequency was similar the cutaneous variants (7/17, 41.2%). (Table 2).

**Table 2:** KIT mutation frequency in BRAF/NRAS double wild type melanoma

	Number of cases	KIT mutation rate (n,%)
cutaneous melanoma	79	34/79 (43.0%)
UV-induced forms	45	14/45 (31.1%)
ALM	34	20/34 (58.8%) p = 0.014
Mucosal melanoma	17	7/17 (41.2 (*n* = 8)%)

*ALM* acrolentiginous melanoma, UV-induced forms = LMM (lentigo maligna), NM (nodular melanoma), SSM (superficial spreading melanoma), NOS (not otherwise specified). Analysis for significance was done by χ2 test

In the 34 KIT mutant skin melanoma cases 38 mutations have been detected because in two cases double mutations and in one case triple mutations occured. Analysis of mutations of skin melanoma in KIT exons indicated that, similar to GIST, exon 11 is the most frequently involved one (44.7%) followed by exon 9 (21.1%), exon 17 (15.8%) and exon 13 (13.2%) and exon 17 (13.2%) (Table 3) There were no significant differences between the UV- and non-UV melanomas in case of exon-9 and exon-11 involvements but exon 18 mutations found in UV melanoma exclusively and exon 13 and 17 mutations were more prevalent in non-UV melanomas. On the contrary, in mucosal melanoma the most frequently involved exon was exon 9 (37.5%) followed by exon 13 and exon 17 (25%, each) while exon 11 was the least frequent. (Table 3) In this form of melanoma also double mutations of KIT exons occured in one case.

**Table 3:** Involvement of KIT exons in melanoma

KIT exon	9	11	13	17	18
cutaneous (*n* = 38)	8/38 (21.1%)	17/38 (44.7%)	5/38 (13.2%)	5/38 (13.2%)	3/38 (7.9%)
UV-induced (*n* = 15)	3/15 (20.0%)	7/15 (46.7%)	1/15 (6.7%)	1/15 (6.7%)	3/15 (20.0%)
ALM (*n* = 23)	5/23 (21.7%)	10/23 (43.5%)	4/23 (17.4%)	4/23 (17.4%)	0
mucosal (*n* = 8)	3/8 (37.5%)	1/8 (12.5%)	2/8 (25%)	2/8 (25%)	0

*ALM* acrolentiginous melanoma

Analysis of the mutational hotspots identified exon 9 codon 482/491/492, exon 11 codons 559/570/572, exon 13 codon 642, exon 17 codon 822 and exon 18 codon 853 as recurrent sites. It is of note that mutations in the nearest codons of such hot spots were also found to be clustered in KIT mutant melanomas (Table 4).

**Table 4:** KIT codon involvement in mutations in melanoma

KIT exon	9	11	13	17	18
UV-induced, skin (n = 15)	c459 c465 c471	c551 c558-561del c559 (2x) c566 c573-584del c575-581del	c641	c816	c847 c853 (2x)
ALM (n = 23)	c482 c491 (2x) c492 (2x)	c557-561del c559 (2x) c570-576del (4x) c572 (2x) c576	c642 (2x) c643 c657	c815 c818 c822 (2x)	
mucosal (n = 8)	c451 c482 c492	c565	c658 c660	c822 c833	

*ALM* acrolentiginous melanoma, *c* codon

## Discussion

There are no published data on the molecular epidemiology of skin melanoma from the central European region. Here we show, that similar to other geographical regions, the BRAF mutation rate is predominant (45.4%). In our cohort, as well as in the one published recently [12], NRAS mutations rate was found to be 20%, a similar rate compared to other geographical regions reported. [5] There is a perception in the literature that KIT mutation rate in skin melanoma is low [6] which is based on the first publication on this issue [4]. However, a recent meta-analysis of publications on KIT mutation frequencies in melanoma demonstrated that the average frequency is 9.5% with great variability [13]. KIT mutation is associated with older age, with CSD melanoma, the mucosal and acrolentiginous forms [13]. As an extreme example, recent data from Slovenia, a neighboring country to Hungary, KIT mutation frequency was found to be 1.3% [11] while an analysis from Italy (UV-and non-UV forms) and France (mucosal only) found ~10% [14, 15]. One explanation for such discrepancies is the composition of the melanoma cohorts: frequently including mucosal melanomas as well. Another pausible explanation for this discrepancy is the testing method: in several studies only GIST-exons have been analysed while in others exons 9 through 18. Our analysis on the Hungarian skin melanoma patients found 14.9% KIT mutation rate, which is higher than worldwide rates, based on analysis of the most important five exons, 9,11,13,17 and 18. It is of importance that our cohort, similar to the Italian one, contained skin melanomas of the UV- as well as the non-UV sites. [15] We show here also, that the KIT mutation rate in double wild type mucosal melanoma in central Europe is comparable to the cutaneous variants. However, it has to be considered that the BRAF/NRAS mutation frequency is very low in mucosal melanoma, therefore the KIT mutation rate must be much higher in mucosal melanoma as compared to cutaneous locations but a statistical analysis cannot be done on our small cohort.

It is another question what the mutation pattern of KIT in melanoma is as compared to the KIT-mutant prototype cancer, GIST. In GIST the vaste majority of cases are characterized by exon 11 mutations (~70%) followed by exon 9 (10%) while other exons are rarely involved [1, 10]. Unfortunately, most of the large melanoma cohorts on KIT mutation did not report completely the exon involvements. In our skin melanoma cohort, similar to GIST, the most frequently mutated KIT exon is exon 11, although at lower frequency than in GIST (44.7%) followed by exon 9 (21.1%) and the other three exons (e13, e17, e18) with significant rates, suggesting a much broader carcinogenic targeting in melanoma. This melanoma KIT mutation pattern is highly similar to the one published from China, except the involvement of exon 9 [7]. Concerning mutational hotspots, in GIST exon 9, codons 502–503 were identified [10] unlike in our melanoma cohort where codons 491/492 were detected. It is of note that in exon 11, both in GIST [10] and in our melanoma patients, codons 557/558 are common targets but nearby hotspot, c559, also exists in skin melanoma. In exon 13 in GIST [10] as well as in melanoma the hotspot is codon 642, but in melanoma the nearby codons are also frequent. This mutational hotspot profile detected in Hungary is highly similar to those detected in China [7]. Similarly, in exon 17 GIST [10] and melanoma share codon 822 as hotspot unlike in exon 18 where GIST is characterized by codon 842 mutation [10] unlike melanoma. These differences and similarities could be important when KIT is used as molecular therapeutic target in melanoma since experience on GIST can be applied to melanoma.

The best clinical experience with KIT inhibitor therapy we have is GIST where exon 11, exon 13 and exon 9 mutations have been shown to confer sensitivity to imatinib and sunitib [1, 10, 16]. In KIT mutant AML sensitivity to KIT inhibitors is unknown for exon 17/c816 mutations [3, 10, 16]. In KIT mutant thymic carcinoma exon 9/c490, exon 11/c553, c557, c559, c576 confers sensitivity, while exon 17/c820 mutation caused resistance to KIT inhitors [16]. In melanoma there are three trials with KIT inhibitors. Partial responses have not been observed in exon 9 mutant melanomas [17–19]. On the other hand, responses were regularly detected in melanoma having exon 11 c576, c577, c557, c559, c560 mutations [17–19]. Furthermore, partial response was detected also in exon 13 c642 mutant melanoma in all the three trials [15–17]. Since more than half of the KIT mutant melanomas harbor exon 11 and exon 13 mutations, this is a significant patient population which could be treated with KIT inhibitors [20]. Considering the relatively high mutation rate of KIT in melanoma, as compared to other mutated oncogens of other solid tumors where target therapy is available (see for example lung adenocarcinoma), it seems to be mandatory to screen BRAF/NRAS double wild type melanoma patients for KIT mutations at least in exon 11/13, irrespective of the type of melanoma.

## Abbreviations

ALM: Acrolentiginous melanoma
AML: Acute myeloid leukemia
c: Codon
CSD: Chronic sun damage
GIST: Gastrointestinal stroma tumor
LMM: Lentigo maligna melanoma
NM: Nodular melanoma
pts: Patients
SSM: Superficial spreading melanoma
